# Nutritional supplement use by elite young UK athletes: fallacies of advice regarding efficacy

**DOI:** 10.1186/1550-2783-5-22

**Published:** 2008-12-15

**Authors:** Andrea Petróczi, Declan P Naughton, Gemma Pearce, Richard Bailey, Andrew Bloodworth, Michael McNamee

**Affiliations:** 1 Faculty of Science, Kingston University, Penrhyn Road, Kingston upon Thames, Surrey, KT1 2EE, UK; 2 School of Sport & Exercise Sciences, University of Birmingham, Edgbaston, Birmingham, B15 2TT, UK; 3 School of Education, University of Birmingham, Edgbaston, Birmingham, B15 2TT, UK; 4 Department of Philosophy, Humanities and Law in Healthcare, School of Health Science, Swansea University, Swansea, SA2 8PP, UK

## Abstract

**Background:**

The objective was to study nutritional supplement use among young elite UK athletes to establish whether a rationale versus practice incongruence exists, and to investigate the sources of information. Survey data were analysed for association between supplements used and motives for using such substances among young athletes along with the sources of advice and literature precedents on supplement effects.

**Methods:**

Participants were elite UK male and female athletes, within the age range between 12 and 21 (n = 403), mean age 17.66 ± 1.99. Associations between type of supplements and reasons for using supplements were tested by calculating Pearson's χ^2 ^and the strength of these symmetric associations shown by phi (ϕ) association coefficients.

**Results:**

Single supplement use was reported by 48.1%, with energy drinks being the most popular, consumed by 41.7% of all athletes and 86.6% of the supplement users in the sample. No agreement was observed between athletes' rationale and behaviour in relation to nutritional supplements except for creatine. Among health professionals, nutritionists and physiotherapists, followed by coaches, were most frequently consulted. Answers regarding reasons and supplements used showed incongruence and suggest widespread misinformation regarding supplements and their effects is an issue for the young athlete.

**Conclusion:**

Widespread supplement taking behaviour was evidenced in the young elite athlete population with the most notable congruence between rationale and practice among young athletes being performance-related. Young athletes in the present sample appear to be less 'health conscious' and more 'performance focused' than their adult counterparts. Further research, using a full list of supplements, is warranted to test the hypothesis that health consciousness is less dominant in supplement choice by young athletes.

## Background

'Supplement' is an overarching name for vitamins, minerals, herbal remedies, and other substances taken orally and regulated as foods and are subject to the general provisions of the Food Safety Act 1990, the Food Labelling Regulations 1996 and the Trade Descriptions Act 1968. In the UK, supplements are required to exhibit efficacy before marketing only if they contain medical claims and fall outside food regulations. Previous studies have highlighted incongruencies between choices of supplement use in adult high performing athletes and reasons for use. Choices of supplement for maintenance of health and performance enhancement were divergent from informed choices based on scientific evidence [1,2].

In contrast to the adult population, young developing athletes have different nutritional requirements which are critical to maintain growth, health and to achieve athletic potential [3]. A recent study of 32 track and field junior athletes selected for Team Great Britain at the World Junior Championships found that 62 per cent of the sample used supplements [4]. Females (75%) were found to use more supplements than males (55%) although this difference was not statistically significant. This trend, also found elsewhere [5], may be attributed to a greater knowledge of nutritional needs among females, greater genuine need for supplementation (e.g. menstrual loss) or advertising campaigns having a greater influence on females [4]. Multivitamins were used most frequently, followed by vitamin C and then iron [4,6]. As reviewed by McDowall [7], seven studies over a twelve year period report the prevalence of supplement use between 22 and 71% in young athletes (age ranges from 13–19). Health benefits, illness prevention, enhancing performance, taste, rectifying a perceived poor diet and increasing energy were the most frequently cited reasons for supplement use among young athletes [7,8].

## Aims

Supplement use appears to be widespread in young athlete populations, for both health reasons and for the enhancement of performance. The aim of this paper is to better understand nutritional use among elite young athletes, specifically exploring (i) sources of advice, and (ii) incongruence between the rationale for use and the supplements used, with a view to enhance sports coaches and health practitioners' understanding of supplement use in the young athlete. Results will be compared to those obtained from a similar survey among adult high performing UK athletes [1,2].

## Methods

### Measures

Questions used for this analysis were from the modified version of the original UK Sport "Drug-Free Sport" survey [9]. The modified version of the questionnaire was piloted in sub-elite sports groups, and sent to sports coaches for detailed feedback. In light of the age range of this population, it was predictable that the suggested changes focused on the simplification of certain technical terms, as well as the general accessibility of the language used. The questions used for the analysis in this paper were: i) 'If you use/have used any of the following supplements, who advised you to take it?' with responses recorded on a ten by ten matrix of supplements and advisors; ii) 'How much do you agree or disagree with the following statement: 'You have to take supplements to be successful'; with answers recorded on a 4-point Likert-type scale with the fifth option being 'do not know' instead of the neutral mid-point; and iii) 'Which of the following (if any) describe why you take a supplement or supplements?' Response options are shown in Tables 1 and 2. It must be noted that athletes were not explicitly asked to give a rationale for using a particular supplement. Congruency was statistically tested between two answers athletes gave independently by chi-square test of association and the strength of relationship was estimated by calculating *ϕ *coefficients [10]. Results are presented as *chi-square *statistics (*χ*^2^), *ϕ *coefficients and their corresponding *p*-values indicating statistical significance.

**Table 1:** Summary of pairwise associations between health maintenance reasons for use and type of supplements used.

**Nutritional supplements^a^**	**Avoid sickness**	**Imbalanced diet**	**Lack of sleep**	**Overcome injury**	**Aid recovery**
**Multivitamin**	χ^2 ^= 3.684	χ^2 ^= .060			χ^2 ^= 21.763
	ϕ = .096	ϕ = .012	NA	NA	ϕ = .233
	(.055)	(.806)			**(< .001)**
**Echinacea**	χ^2 ^= .726	NA			
	ϕ = .042		NA	NA	NA
	(.394)				
**Vitamin C**	χ^2 ^= 4.417	χ^2 ^= .021		χ^2 ^= .261	
	ϕ = .105	ϕ = .007	NA	ϕ = .015	NA
	(.036)	(.886)		(.610)	
**Iron**	NA	χ^2 ^= .568			
		ϕ = .038	NA	NA	NA
		(.451)			
**Ginseng^b^**	χ^2 ^= 0.593		χ^2 ^= 17.866		
	ϕ = -.038	NA	ϕ = .211	NA	NA
	(.441)		(< .001)		
**Whey protein**		χ^2 ^= 1.957			
	NA	ϕ = .038	NA	NA	NA
		(.162)			
**Caffeine**			χ^2 ^= 3.255		
	NA	NA	ϕ = .090	NA	NA
			(.071)		
**Creatine**		χ^2 ^= 1.995			
	NA	ϕ = -.070	NA	NA	NA
		(.158)			
**Energy drinks**		χ^2 ^= 4.765	χ^2 ^= .137		
	NA	ϕ = -.109	ϕ = -.018	NA	NA
		(.029)	(.711)		

^a ^melatonin excluded from the analysis owing to insufficient data (n = 4)

^b ^expected association from anecdotal effect.

Each cell displays the *χ*^2 ^coefficient, *ϕ *and the corresponding p-values in parentheses (NA = no association expected).

**Table 2:** Strength of pairwise associations between performance enhancing reasons to use and type of supplements used.

**Nutritional supplements^a^**	**Maintain strength**	**Enhance endurance**	**Able to train longer**
**Vitamin C**			χ^2 ^= 6.430
	NA	NA	ϕ = .126
			(.011)
**Iron**		χ^2 ^= 5.275	
	NA	ϕ = .115	NA
		(.022)	
**Ginseng**^b^			χ^2 ^= 4.518
	NA	NA	ϕ = .106
			(.034)
**Whey protein**	**χ^2 ^= 94.355**		**χ^2 ^= 12.723**
	**ϕ = .484**	NA	**ϕ = .178**
	**(< .001)**		**(< .001)**
**Caffeine**		χ^2 ^= 11.170	χ^2 ^= 2.536
	NA	ϕ = 0.167	ϕ = .079
		(.001)	(.111)
**Creatine**	**χ^2 ^= 80.327**		
	**ϕ = .447**	NA	NA
	**(< .001)**		
**Energy drinks**	NA	**χ^2 ^= 44.895**	**χ^2 ^= 19.224**
		**ϕ = .334**	**ϕ = .129**
		**(< .001)**	**(< .001)**

^a ^melatonin excluded from the analysis owing to insufficient data (n = 4); performance enhancing effect is not expected for multivitamin and echinacea

^b ^expected association from anecdotal effect.

### Ethical approval

Ethical procedures followed established practices [11,12]. Ethical approval was granted by the Research Ethics Committee of the School of Health Science, University of Wales Swansea. Consent forms were sent to all participants (and parents/guardians where proxy consent was necessary), providing details of the aims and methods of the study, and explaining that their participation was voluntary and that any information given would remain anonymous and confidential and secured with the research team only. For those participants under the age of sixteen, consent forms were directed towards their parents/guardians, and additional *assent *forms were sent to the young participants. In light of the nature of this study, all data were anonymised.

### Analyses

**Figure 1:**
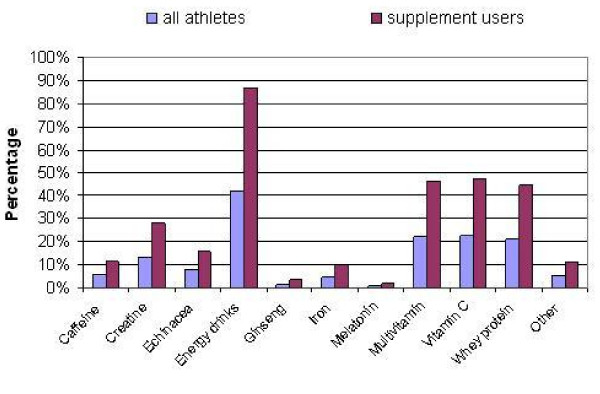
Supplement use reported by young (age 12–21) elite athletes; n = 403 (all athletes) and n = 194 (supplement users); each equates to 100%.

**Figure 2:**
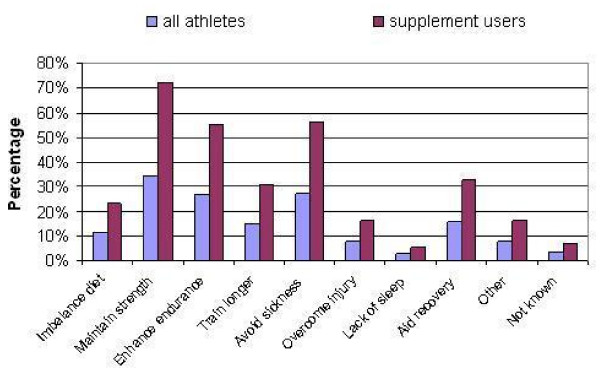
Reasons for supplement use reported by young (age 12–21) elite athletes; n = 403 (all athletes) and n = 194 (supplement users) each equates to 100%.

Responses were entered, coded and analysed using a specialist statistical analysis software package (SPSS 15.0 for Windows). The level and distribution of use of each supplement and reasons for using supplements were shown as frequency counts and percentages (Figures 1 and 2). Association between type of supplements and reasons for using supplements were examined by calculating chi-square test statistics and phi coefficients, and presented along with their corresponding p-values (Tables 2 and 3). In line with previous reports on the adult data [1,2], cells have been identified as 'expected congruency between reasons and actions' based on empirical evidence (expected correct association) using published research on the effects of nutritional supplements [13-24]. Bar charts were used to illustrate athletes' agreement regarding supplements.

**Table 3:** Relative percentage of congruent answers by rationale for supplement use and supplement used by athletes.

		**Maintain strength**				**Enhance endurance**			
		**Yes**		**No**		**Yes**		**No**	
**Whey**	**Yes**	68	48.6%	18	6.9%				
**protein**	**No**	72	51.4%	244	93.1%				
		140	100.0%	262	100.0%				

**Creatine**	**Yes**	48	34.3%	6	2.3%				
	**No**	92	65.7%	256	97.7%				
		140	100.0%	262	100.0%				

**Energy**	**Yes**					74	69.2%	94	31.9%
**drinks**	**No**					33	30.8%	201	68.1%
						107	100.0%	295	100.0%

### Participants

Young elite athletes with ages ranging between 12 and 21 were identified via the Talented Athlete Scholarship Scheme (TASS); the UK Sport World Class Development Programme (WCDP); the National Academy Systems in cricket and Rugby Union; and via the Professional Footballers Association.

The questionnaire was distributed to 1,674 athletes. For TASS athletes, questionnaires were administered by TASS programme staff; for WCDP athletes, cricket and rugby union academy members and young professional footballers by the research team. Questionnaires were delivered to the athletes by post to their home address with a consent/assent form and information sheet. A period of one month was given for the return of the questionnaires. Follow up letters were sent to the athletes after this period to ask them to fill out the questionnaire if they had not already sent it back. A Freepost envelope was included with the questionnaire.

## Results

Of the 1,674 questionnaires distributed, 412 were returned and 403 were within the age range required. The highest response rate was from WCDP athletes (43%), followed by TASS (29%) and National Government Bodies (NGB) athletes (18%), calculating an overall response rate average of 25%. Overall this average was brought down due to a very low response rate from the football players in the NGB athlete group. The sample predominantly consists of white (93.8%) male (64.5%) athletes compared to females (33.0%) with 2.5% of the information missing. Over two thirds of respondents (67.0%) are between age 16 and 19 whilst the 12–16 and the 20–21 age groups formed 12.5% and 19.9%, respectively. Age information for 0.5% of the participants was missing. The mean age was 17.66 ± 1.99.

Close to one-third of the athletes in the sample were rugby union players (27.8%), followed by football players (13.9%) and swimmers (6.7%). Table tennis, equestrian, fencing, synchronised swimming, rowing, diving, water polo, canoeing, golf, judo, orienteering, taekwondo, tennis, cricket and disability athletics were contributed to the sample by 1 to 5%, listed in decreasing order. The individual contribution from the remaining 17 sports was ≤ 1%. The majority of the young athletes (78.4%) did not believe that nutritional supplementation was necessary to be successful in sport with only 4.2% claiming not having the knowledge to answer the question. However, contrary to this belief, almost half of the respondents (48.1%) reported using at least one supplement from the list provided (Figure 1) with a considerable proportion of athletes using more than one supplements. On average, 2.96 supplements were used among the elite young athletes (574 instances with 194 supplement users).

Figure 1 shows the proportion of athletes in the sample and among those who reported supplement use. The most popular supplements were: energy drinks (41.7% of all athletes and 86.6% of supplement users reported), followed by vitamin C (22.8% and 47.4%), multivitamin (22.8% and 47.2%), whey protein (21.3% and 44.3%), creatine (13.4% and 27.84%), echinacea (7.7% and 16.0%), caffeine (5.7% and 11.9%), iron (4.7% and 9.8%), ginseng (1.7% and 3.6%) and melatonin (1.0% and 2.1%). Among other supplements, glutamine/glucosamine, branch-chain amino acid, cod liver oil, B vitamin complex, Maximuscle, Met-RX, norateen, carbohydrates and protein shakes, omega oils and probiotics were listed.

Among the desired outcomes resulting from supplement use, maintaining strength was the most frequently cited reason among all athletes in the sample (34.7%) and supplement users (72.2%), followed by avoiding sickness (56.1% of users) and endurance enhancement (55.2% of users). One-third of the supplement user athletes listed the ability to train longer (30.4%) and helping to recover (32.5%) among the reasons, whereas 23.2% take supplements to remedy imbalanced diet (Figure 2).

Overcoming injury and other reasons equally contributed with 16.5% to the picture. Given the age group, an unexpectedly modest proportion (5.7%) reported lack of sleep among the reasons, whereas 13 athletes (6.7%) claimed that they do not know why they take supplements.

**Figure 3:**
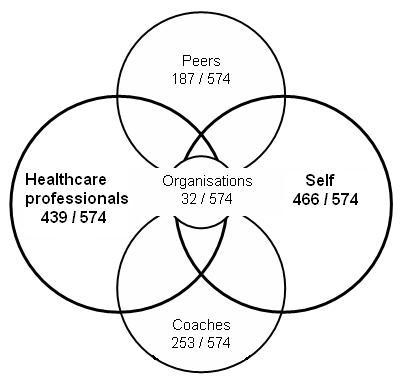
Source of information regarding the supplements reported, expressed as ratio between the number of sources and incidents reported.

With regard to sources of advice on nutritional supplementation, many young athletes appear to decide on their nutritional supplementation themselves without advice. The considerable overlap between self-managed supplementation and medical advice (Figure 3) is, however, somewhat reassuring. The coaches' role in advising athletes on supplements, especially in taking energy drinks and protein was also evidenced. Among health professionals, athletes indicated that advice was sought from nutritionists and/or physiotherapists. The only exception to this pattern was iron supplementation, which was taken following general practitioners' advice.

To assess athletes' understanding of the benefits of nutritional supplements and to investigate the presence or lack of informed choices, the association between used supplements and reasons for using supplements were tested. Under an assumption that the choices of athletes were informed ones, a strong association is expected between a given reason for their use and a given supplement. The most relatively strong associations fell in the performance quadrant using creatine, whey protein and energy drinks (Tables 1 and 2). Notably, the strongest associations were found between maintaining strength and whey protein (*χ*^2 ^= 94.355, *ϕ *= .484, *p *< .001) and creatine (*χ*^2 ^= 80.327, *ϕ *= .447, *p *< .001).

Among health maintenance reasons, only multivitamin showed a considerable congruence with aiding recovery. Interestingly, no association between vitamin C and health maintenance was evidenced.

Table 3 shows the agreement expressed as percentages between rationale and practice. In an ideal situation, assuming that all athletes are fully informed, the Yes-Yes quadrant (i.e. using a substance) should be 100%. A high percentage in the mixed cells (YN and NY) indicates lack of knowledge or potentially random choice of supplements. Compared to this best case scenario, the best agreement between rationale and practice was found for energy drinks and endurance enhancement. 69.2% of the respondents reported using energy drinks when endurance enhancement was listed among the reasons for taking supplements. As for creatine and whey protein, among those who claimed to use supplements to maintain strength, only 34.4% actually reported using creatine and 48.6% used whey protein. Considering that all athletes in the sample were supplement users, these athletes used supplements other than those that would help to achieve their expressly stated outcome (e.g. increased strength).

**Figure 4:**
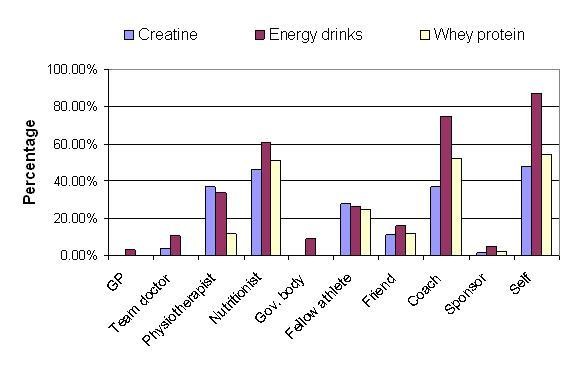
Source of advice selected for taking creatine (54), energy drinks (168) and whey protein (86) among supplement user young athletes (n = 194).

Figure 4 further investigates the role of various formal and informal support personnel in the use of creatine, protein and energy drinks. As expected, the majority of the supplement user athletes reported the consumption of energy drinks based on their own decisions, although a relatively high proportion of athletes reported that coaches also advised them on these drinks. On the contrary, sponsors' contributions were unexpectedly low. Congruent with the overall picture, general practitioners and team doctors appear to be less influential than physiotherapists, coaches or nutritionists. This result clearly warrants further qualitative research.

## Discussion

Recently, considerable attention has been given to the benefits, and to a lesser degree, drawbacks of nutritional supplement use. The 2007 report of the Science and Technology Committee of the House of Commons, among other important recommendations, urges researchers to explore acceptable means to increase human performance. Under the strict regulation of the World Anti-Doping Agency (WADA) and their expanding list of prohibited substances, athletes and researchers have turned to nutritional supplements: vitamins, minerals, herbs, botanicals and non-herb non-mineral substances. A study commissioned by UK Sport evidenced that supplement use is predominant among adult high-performing UK athletes (62%) with an average of 3.22 supplements used per athlete [25]. In comparison, only 48.1% of emerging UK elite athletes reported supplementation in the present study with an average of 2.96 supplements used.

The use of multiple supplements is a common practice which manifests in both a higher number of adverse reactions and in those reactions being more severe [26,27]. Whilst the potentially adverse effects of long term use of supplements and concomitant use are yet to be established for many substances, the reported multiple use of supplements, coupled with self-managed supplementation is concerning. The exceptions are caffeine (for example the effects on the cardiovascular system) and vitamin C (for example as a pro-oxidant intake at high levels), where the benefits and side effects from long term use have been established [28,29].

The supplement-use profiles for both adults and young athletes contained multivitamin and vitamin C among the most widely used supplements. Moreover, adult athletes showed the highest level of informed choices in health maintenance reasons and supplements, emerging athletes exhibited more congruency in performance enhancements [1,2]. For example, contrary to the adult data, which showed no association between whey protein and strength, the association between protein and strength was the strongest one among young athletes. Among all the supplements queried among UK athletes, creatine and whey protein use can be the most accurately predicted from the reasons given for using such supplements and vice versa, complemented by vitamin C in the adult survey and energy drinks in the present youth survey. With the exception of these substances, there was a considerable mismatch of rationale and practice. The appearance of the energy drinks in the youth data is in keeping with the literature [30]. In addition to energy drinks, creatine and whey protein, young athletes used a wide variety of supplements seemingly without a clear understanding of what supplements can or cannot do. The significant self-managed supplementation may partially explain the observed lack of congruence between reasons and supplement used.

### Implications

Athletes appear to have a perceived need for nutritional supplementation. Although speculative at this point, based on the existing literature, it is not unreasonable to assume that athletes are likely to take a combination of substances and perhaps in large doses. For example, the OSL for creatine (5 g/day), is routinely exceeded both in practice and in scientific studies [31,32]. Reservations apply to even the most commonly used supplements regarding long term use, combinations and appropriate dosages. These reservations concern: 1) an increased health risk to an otherwise healthy population and 2) the possibility of positive doping tests caused by supplements containing banned substances. To negate potential health risks arising from potentially inappropriate supplement use, an increased involvement of health professionals, with appropriate training, is desirable.

The partial limitation of this study arises from using a closed list of nutritional supplements. Whilst the wide range of supplements include both relatively harmless supplements (e.g. energy drinks, bars), through macro- and micronutrients to potentially harmful substances (e.g. some herbs), the survey was limited to ten relatively harmless substances. However, the aim of this study was to investigate the congruence between rational and practice. Therefore this paper does not provide a comprehensive picture of supplement use patterns and interpretation along this line must be done cautiously. The list of 'other' substances indicates that the range of supplements used regularly expands well beyond the ten substances investigated. As dosage and/or concomitant use influence toxicity, having information of these parameters is paramount. Future research should also expand the list of supplements and include other supplements with particular emphasis on those that are potentially harmful and those with unknown effects (e.g. herbal extracts or exotic supplements).

## Conclusion

Widespread supplement taking behaviour was evidenced in the young elite athlete population. Contrary to the result from the adult survey [1,2], the most notable congruence between rationale and practice among young athletes was performance-related. This observed difference in supplement-using patterns, may partially be explained by age differences. Young athletes in the present sample appear to be less 'health conscious' and more 'performance focused' than their adult counterparts. Further research, using a full list of supplements, is warranted to test the hypothesis that health consciousness is less dominant in supplement choice by young athletes.

## Competing interests

AP and DPN declare no conflicting interest. The project from which these data emerged was funded by UK Sport with MMcN as PI. MMcN and RB have been supported by UK Sport's international fund to attend international symposia during the last 5 years. MMcN has received consultancy for research ethics from UK Sport during the last year.

## Authors' contributions

MMcN, RB and AB designed the study, developed the questionnaire and collected the data. GP contributed to data collection and prepared the data set for statistical analysis. AP and DPN carried out the analysis and drafted the manuscript. All authors read and approved the final manuscript.
